# Investigation of Particle Rotation Characteristics and Compaction Quality Control of Asphalt Pavement Using the Discrete Element Method

**DOI:** 10.3390/ma17112764

**Published:** 2024-06-05

**Authors:** Zhi Zhang, Hancheng Dan, Hongyu Shan, Songlin Li

**Affiliations:** School of Civil Engineering, Central South University, Changsha 410075, China; zhizhang@csu.edu.cn (Z.Z.); shanhy@csu.edu.cn (H.S.); 234811173@csu.edu.cn (S.L.)

**Keywords:** asphalt pavement, vibratory compaction, rotational characteristic, compaction quality, discrete element method

## Abstract

The compaction of asphalt pavement is a crucial step to ensure its service life. Although intelligent compaction technology can monitor compaction quality in real time, its application to individual asphalt surface courses still faces limitations. Therefore, it is necessary to study the compaction mechanism of asphalt pavements from the particle level to optimize intelligent compaction technology. This study constructed an asphalt pavement compaction model using the Discrete Element Method (DEM). First, the changes in pavement smoothness during the compaction process were analyzed. Second, the changes in the angular velocity of the mixture and the triaxial angular velocity (TAV) of the mortar, aggregates, and mixture during vibratory compaction were examined. Finally, the correlations between the TAV amplitude and the coordination number (CN) amplitude with the compaction degree of the mixture were investigated. This study found that vibratory compaction can significantly reduce asymmetric wave deformation, improving pavement smoothness. The mixture primarily rotates in the vertical plane during the first six passes of vibratory compaction and within the horizontal plane during the seventh pass. Additionally, TAV reveals the three-dimensional dynamic rotation characteristics of the particles, and the linear relationship between its amplitude and the pavement compaction degree aids in controlling the compaction quality of asphalt pavements. Finally, the linear relationship between CN amplitude and pavement compaction degree can predict the stability of the aggregate structure. This study significantly enhances quality control in pavement compaction and advances intelligent compaction technology development.

## 1. Introduction

The compaction of asphalt pavement is a critical step in ensuring its service life and durability [1]. Asphalt mixtures and other civil engineering materials exhibit complex mechanical properties [2,3], and traditional compaction methods lack effective real-time quality assessment, which can lead to uneven compaction (either undercompaction or overcompaction) [4]. Such unevenness can result in early pavement damage or aggregate breakage [5,6], thereby affecting the performance and stability of the asphalt pavement. Therefore, effective control of compaction quality is key to enhancing pavement performance.

Emerging intelligent compaction technology, equipped with integrated measurement systems, satellite-based navigation systems, thermal imaging devices, motion sensing equipment, and other tools [7], aims to overcome the limitations of traditional asphalt pavement compaction methods to achieve superior compaction quality. This technology reflects the compaction status of the entire pavement structure rather than individual asphalt surface courses, resulting in a weak correlation between intelligent compaction measurements and asphalt surface course density [8]. Therefore, it is necessary to delve into the compaction mechanism of asphalt pavements, particularly from the perspective of particle dynamics and rotational behavior. Particles within asphalt mixtures reduce voids by moving and spinning throughout the compaction phase, thereby enhancing the load-bearing capacity of the pavement [9]. Hence, studying the rotation and movement of asphalt mixture particles during compaction is crucial for understanding the compaction mechanism of asphalt pavements and further optimizing intelligent compaction technology.

In recent years, SmartRock sensors have been extensively used to monitor the rotational characteristics of asphalt mixture particles during compaction processes [10,11]. Wang et al. [12] studied the rotational features of aggregates during gyratory compaction using SmartRock, correlating it with the density of asphalt mixtures. Moreover, the study of particle rotational characteristics using SmartRock provided effective indicators for researching the compaction of asphalt mixtures [9]. However, due to the limitations in the number and placement of sensors within the mixture, their data may not represent the overall compaction condition of the asphalt mixture, affecting the universality of the research findings. Currently, some researchers use Finite Element Method (FEM) and Discrete Element Method (DEM) to investigate the compaction characteristics of asphalt mixtures at both macroscopic and microscopic scales [13]. Koneru et al. [14] simulated the gyratory compaction of asphalt mixtures using the finite element software Abaqus. Sun et al. [15] developed a three-dimensional finite element simulation for vibratory compaction of asphalt pavements using Abaqus, analyzing the stress distribution beneath the wheel. Masad et al. [16] simulated the compaction process of asphalt mixtures under rolling conditions using finite element programs. Although the FEM has made some progress in simulating the compaction of asphalt mixtures, it still cannot accurately simulate the heterogeneity and particle motion characteristics of these mixtures [17].

The FEM focuses on reducing voids through the extrusion of aggregates and colloids, while DEM emphasizes achieving denser packing through the translation and rotation of particles [18,19]. Several studies have utilized the DEM to simulate the compaction process of asphalt pavements. Khateeb et al. [20] used the DEM to model the compaction of porous asphalt mixtures, aiding in understanding the mesostructural changes in the mixture during compaction. Liu et al. [21] established a discrete element compaction model for steel bridge decks to investigate the segregation characteristics of the mixture during compaction. Man et al. [22] proposed a dual-scale discrete element model for simulating the compaction process of a hot asphalt mixture and successfully captured the influence of particle size distribution on the compaction behavior. Chen et al. [23] analyzed the void distribution in the asphalt mixture during compaction using the DEM, finding it effective in simulating the internal void structure of the mixture. Olsson et al. [24] developed a novel DEM modeling approach for studying asphalt compaction, this method can reflect the force distribution network within the material. Furthermore, the angular velocity of the aggregate can reflect its rotational motion within the asphalt mixture. Wang et al. [25] employed the DEM to simulate the precompaction of asphalt pavements, proposing a method to assess the precompaction effect of loose mixtures using the average angular velocity of aggregates. They found that the angular velocity of aggregates can assess the compaction effect of asphalt mixtures. However, angular velocity can only describe the rotation of aggregates around a specific direction and cannot measure their three-dimensional motion in space. In contrast, triaxial angular velocity (TAV) provides a comprehensive evaluation of the three-dimensional rotational behavior of mixture particles during the compaction process of asphalt pavement. This is crucial for understanding the internal compaction mechanisms of asphalt mixtures. Finally, the coordination number (CN) of the particles refers to the average number of particles in contact with a single particle. Niu et al. [26] proposed the concept of CN as a metric to evaluate the contact characteristics of skeletal structures. They found that the CN helps assess the stability of the aggregate structure. Despite these advances, the DEM remains in the preliminary exploration stage in simulating the compaction of asphalt mixture, especially in exploring the dynamic rotational behavior of asphalt mixtures during asphalt pavement compaction and their correlation with the quality of pavement compaction.

To summarize, traditional compaction methods lack real-time quality assessment, which leads to uneven compaction and consequently affects the compaction of asphalt pavement. Although intelligent compaction technology allows for real-time monitoring, it still does not adequately reflect the compaction quality of individual asphalt surface courses. Moreover, SmartRock sensors and numerical simulation methods provide new pathways for a deeper understanding of the compaction mechanisms of asphalt mixtures. These technologies have revealed the characteristics of particle rotation and their relationship with compaction quality, yet a full understanding of these relationships requires further exploration. This paper aims to utilize the DEM software EDEM (Enhanced Discrete Element Method) V2020.1, developed by Altair Engineering, Inc. (Troy, MI, USA), to establish a model for asphalt pavement compaction, to investigate the compaction mechanism of asphalt mixtures, and to further investigate the dynamic response of particle rotation during the vibratory compaction process and its correlation with pavement compaction quality. This can help reflect the compaction quality of individual asphalt pavement courses through particle rotation characteristics. Then, this study has significant implications for improving the control of asphalt pavement’s compaction quality and advancing the development of intelligent compaction technology.

## 2. Materials and Methods

### 2.1. Materials

The type of asphalt mixture utilized within the scope of this research was AC-20C, intended for the middle surface course of asphalt pavements, measuring 6 cm in depth. The asphalt mixture consists of SBS(I-D) modified asphalt, limestone aggregate, and limestone mineral powder, with the limestone and mineral powder sourced from a quarry in Chongqing, China. According to the Marshall mix design procedure, the void ratio for the mixture is 4.0%, and its maximum theoretical density is 2.542 g/cm^3^. The fundamental properties of the mixture and the aggregate gradation of AC-20C are detailed in the study by Zhang et al. [27]. Additionally, the specifications of the modified asphalt are shown in Table 1, and the basic technical specifications of the coarse aggregate are listed in Table 2. All key indicators for the asphalt and aggregates comply with the relevant requirements of the Technical Specifications for Construction of Highway Asphalt Pavements (JTG F40-2004) [28].

### 2.2. Field Experiment

The field test was conducted on a section of asphalt pavement on the Zhengxi Expressway in Zunyi, Guizhou Province, China. After the asphalt mixture was laid by the paver, compaction tests were carried out. Within the same pavement area, three compaction test points were selected within a 200 m range in this experiment. The test points were selected within a 3 m distance from the roadside and marked to facilitate measurements by on-site personnel. This project employed the Dynapac CC624HF (Dynapac, Stockholm, Sweden) double drum roller for compaction tasks, which included both static and vibratory compaction modes. Initially, static compaction was executed at the test point by the roller, which was then succeeded by seven sequences of vibratory compaction. The detailed compaction process and the distribution of on-site compaction equipment can be found in previous literature [27]. After each pass of the roller, a non-nuclear density gauge was used to assess the degree of compaction of the asphalt pavement, serving as an indicator for evaluating compaction quality. To ensure the accuracy of compaction measurements, the non-nuclear density gauge should be securely placed at the test points, ensuring good contact with the pavement and avoiding any movement or vibration.

## 3. Discrete Element Simulation

### 3.1. Model Construction

**Figure 1:**
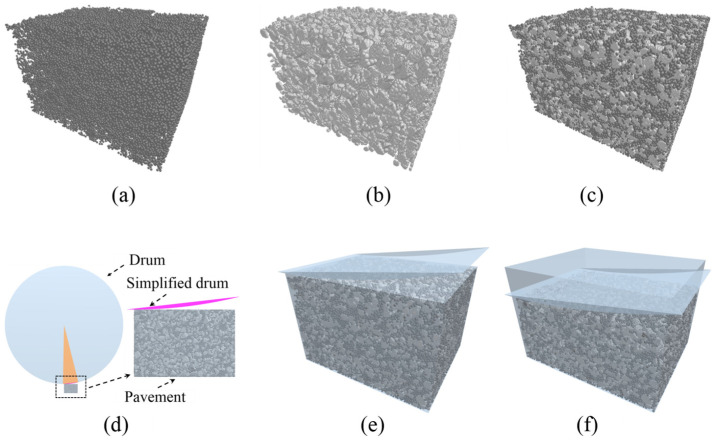
Asphalt pavement compaction model: (**a**) mortar, (**b**) aggregate, (**c**) loose pavement, (**d**) simplified drum, (**e**) pavement before precompaction of the paver, and (**f**) pavement after precompaction of the paver.

The compaction performance of an asphalt mixture is primarily influenced by its microstructure. To enhance the computational efficiency of the model, the asphalt mixture model simplifies the mixture of fine aggregates with a particle diameter smaller than 2.36 mm, along with mineral filler and bituminous binder into an asphalt mortar and simulates it using particles of a uniform radius of 1 mm, as shown in Figure 1a. The asphalt mixture model represents aggregates using irregularly shaped clumps and generates aggregates of various particle sizes based on the aggregate gradation as shown in Figure 1b. A loose asphalt mixture discrete element model composed of asphalt mortar and aggregates is shown in Figure 1c.

Due to the limitations of discrete element computational efficiency, large-scale asphalt pavement compaction simulations face challenges. Therefore, a small-scale model dimension (length 0.15 m, width 0.12 m, height 0.06 m) is used for the simulation. Given that the drum diameter of the roller is considerably larger than the model size, the model’s drum is simplified, as shown in Figure 1d, to avoid additional computational burden. During the simulation, the model used a simplified drum to precompress the loose asphalt mixture within a geometry with dimensions (length 0.15 m, width 0.12 m, height 0.1 m), simulating the compaction state of the pavement after compaction by the paver as shown in Figure 1f. During the loading period, the top drum applies a load to the top of the model. Furthermore, models of three different aggregate distribution states, based on the same asphalt mixture gradation, are calculated to study their reliability.

### 3.2. Mesoscopic Contact Model

**Figure 2:**
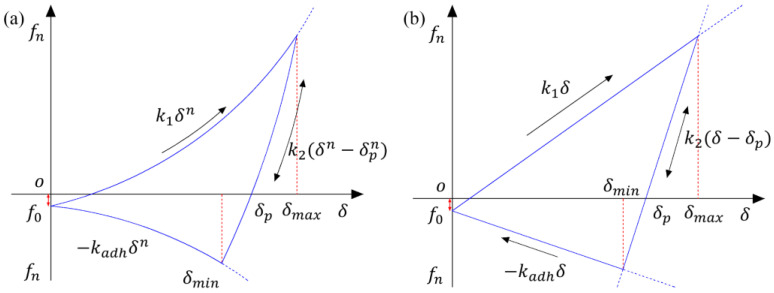
The curve of normal contact force vs. overlap for the contact model: (**a**) nonlinear and (**b**) linear.

The Edinburgh Elastoplastic Adhesion (EEPA) contact model concerns the elastoplastic deformation of particles or aggregates under compressive forces. This model assumes that as the plastic contact area increases, the pull-off strength correspondingly rises, considering the nonlinear relationship between elastic–plastic contact deformation and adhesive contact area. Figure 2 displays the relationship curve between the normal contact force and the particle overlap quantity (f_n_−δ).

The description of loading, unloading, reloading, and the adhesion branches of the model involves five parameters: initial loading stiffness k_1_, unloading and reloading stiffness k_2_, constant adhesion strength f_0_, adhesion stiffness k_adh_, and stiffness exponent n. In the initial phase, the normal contact force follows the initial loading path k_1_. Upon starting to unload, the force switches to the unloading/reloading path k_2_. During reloading, the contact force first increases along path k_2_ until it reaches the maximum loading force, then switches back to the k_1_ path. Furthermore, unloading along the k_2_ path below the plastic overlap δ_p_ leads to the development of adhesive force until reaching the maximum adhesive force. If unloading exceeds the maximum adhesion force point, it results in a decrease in normal overlap and adhesion force until complete separation of the contact is achieved (δ = 0). If reloading occurs on the adhesion branch, the normal contact force increases along path k_2_ until it returns to the k_1_ path and continues loading. If k_1_ is set to be equal to k_2_, the contact model is simplified to an elastic contact model. Detailed information on the contact model can be found in the work by Thakur et al. [31].

**Figure 3:**
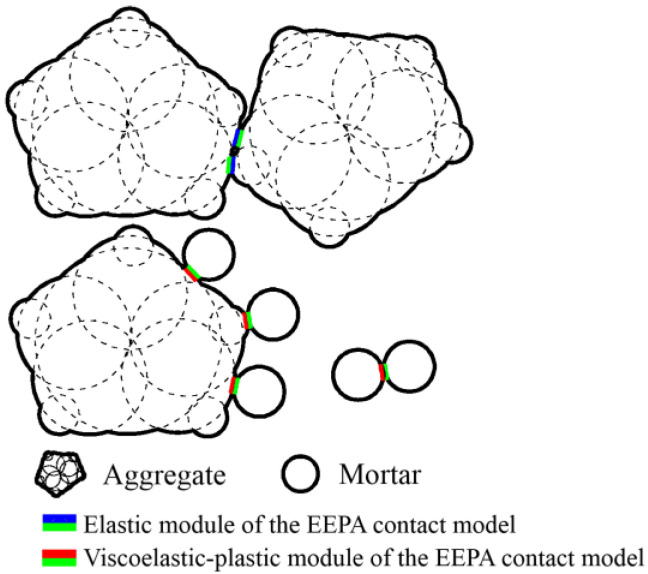
The schematic diagram for the selection of particle contact models.

An asphalt mixture is a viscoelastic and compressible viscoelastic–plastic material at high temperatures. In the model of asphalt pavement, there are three types of contact interfaces: between adjacent aggregates, between aggregates and mortar, and within the mortar itself. The elastic module of the EEPA contact model is used to simulate the elastic properties between adjacent aggregates, while the contact between aggregates and mortar as well as within the mortar adopts the viscoelastic–plastic module of the EEPA contact model to reflect its viscoelastic–plastic behavior during the high-temperature compaction process (see Figure 3). The model parameters include material physical parameters (such as Poisson’s ratio, Young’s modulus, and density) and contact model parameters. The physical parameters of the aggregates are selected based on data provided by the manufacturer. Additionally, the physical parameters of the mortar are based on previous research [32] and are determined through inverse calculations between experiments and simulations, as shown in Table 3.

The parameters of the contact model include constant pull-off force, surface energy, contact plasticity ratio, slope exponent, tensile exponent, and tangential stiffness multiplier. A nonzero value of constant pull-off force indicates the presence of continuous viscous force between particles, and surface energy is used to determine the maximum attractive force between particles. Moreover, the contact plasticity ratio quantifies the plastic deformation between particles, where a value of 0 represents completely elastic behavior. The slope exponent (n) determines whether the model is linear (n = 1) or nonlinear. Lastly, the tensile exponent (X) characterizes the declining trend of the viscous force curve, with X = 1 indicating a linear trend, and larger values indicating a greater decline in the viscous curve. The tangential stiffness multiplier is defined as the proportion between tangential and normal stiffness, whose calculation is detailed in the research by Dan et al. [33]. The values in Table 4 are based on previous studies [21,31] and obtained through inverse calculations between experiments and simulations.

### 3.3. The Vibration Equation of Drum

**Figure 4:**
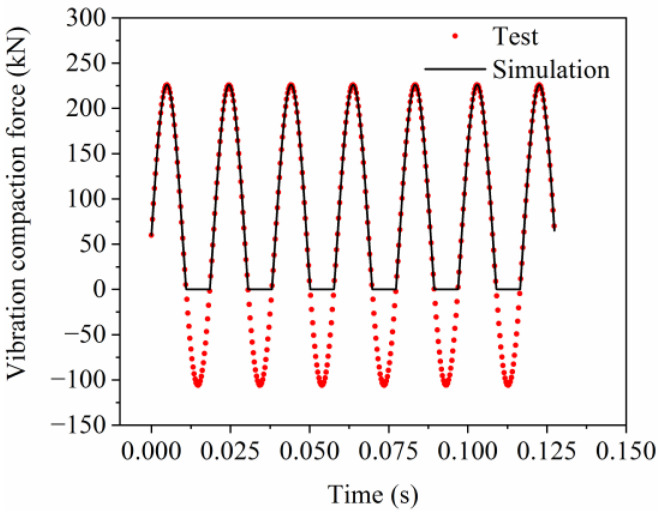
The vibration compaction force of the drum.

When a roller compacts asphalt pavement through vibration, the force primarily originates from the self-weight of the drum and the vertical excitation force produced by the eccentric mass. During the model compaction process, it is essential to ensure that the vibration compaction force is not less than zero, as shown in Figure 4, to prevent separation between the drum and the model. Therefore, the vibration compaction force exerted by the drum on the pavement can be described by Equation (1).

(1) $$F=\left\{\begin{array}{c} 0,\;F<0\\ G+F_{0}sin\left(2\pi ft\right),\;F\geq 0 \end{array}\right.$$

where *F* represents the vibration compaction force, *G* is the self-weight of the drum, *F*_0_ is the excitation force, *f* is the vibration frequency, and *t* is the compaction time. Furthermore, the self-weight of the drum is 60,000 N, the excitation force is 166 kN, and the vibration frequency is 51 Hz.

### 3.4. Simulation Process

To simulate the compaction effects of a double-drum vibratory roller on asphalt pavement, the vibration equation of the drum was employed to model the vibratory compaction action on the pavement. In the field compaction process, the two drums of the roller executed a total of 16 compactions, divided into 9 static drum compactions and 7 vibratory drum compactions. In the model, the compaction process of the drum is conducted strictly in accordance with the onsite compaction process, as shown in Table 5. In a double drum roller, the drum located at the front part in the direction of driving during compaction is referred to as the front drum, whereas the opposite is termed the rear drum. Additionally, the rolling speed of the drum in the model is set at 3.82 km/h, which is consistent with the operating speed of the onsite roller.

### 3.5. Experiment and Simulation Comparison Verification

**Figure 5:**
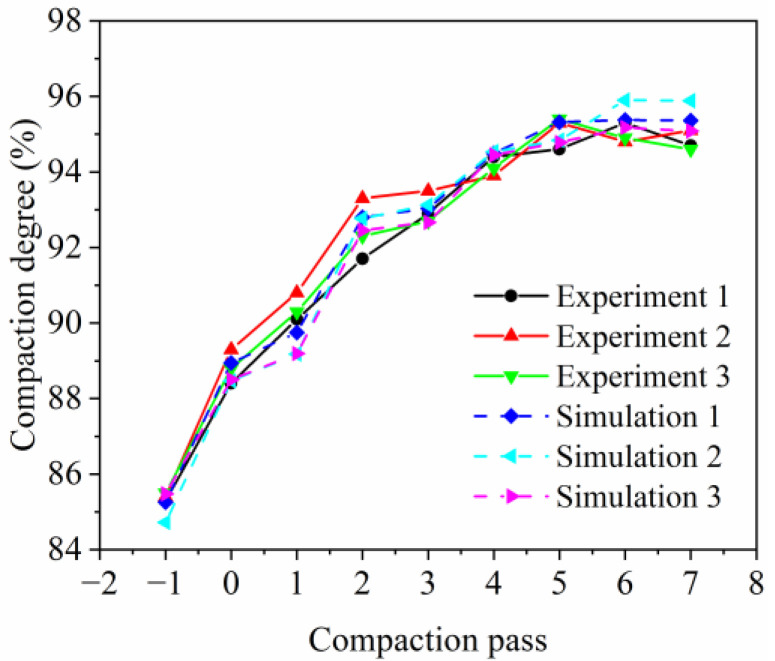
The change in compaction degree with different compaction passes.

The compaction degree of the asphalt pavement changes with the increase in the vibratory compaction pass, as shown in Figure 5. The horizontal axis, with values −1 and 0, respectively, represents the compaction degree after the mixture is paved and after one static compaction pass of the roller. Other numbers indicate the vibratory compaction pass. From Figure 5, it is evident that as the vibratory compaction pass increases, the compaction degree of the asphalt pavement improves, with the trend of changes in the simulation and experiment results being consistent, validating the effectiveness of the simulation.

Table 6 presents the relative error for different compaction passes. The experimental compaction degree for each pass is the average of three sets of experimental data, while the simulated compaction degree is the average of three sets of simulation data after each pass. The relative errors of the simulation results compared with the experimental results are calculated according to Equation (2).

(2) $$Relative\;error=\frac{Simulation-Experiment}{Experiment}\times 100\%$$

As shown in Table 6, the relative error range for different compaction passes is −1.13 to 0.68, indicating that the numerical simulation is relatively accurate. In summary, the discrete element compaction model can effectively simulate the compaction process of asphalt pavements.

## 4. Results and Discussions

### 4.1. Asphalt Pavement Smoothness

**Figure 6:**
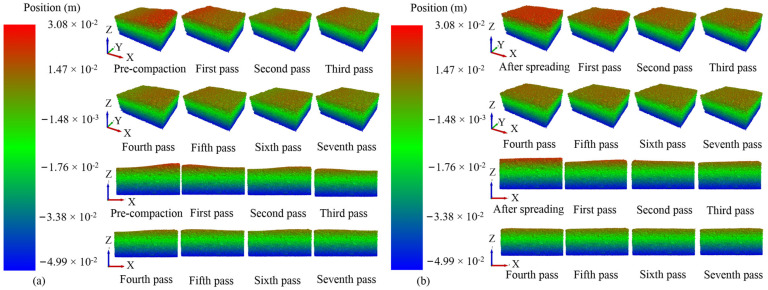
The cloud diagram of the z-axis positions of particles under different compaction passes: (**a**) static compaction and (**b**) vibratory compaction.

During the compaction process, as the compaction pass increases, both the thickness and smoothness of the model change accordingly. Figure 6 displays the reference coordinate system of the model within the computational domain and shows the cloud diagram of the z-axis positions of particles under different compaction passes. There are two working modes of the roller: static compaction and vibratory compaction. Static compaction primarily relies on the weight of the drum itself to compact the asphalt pavement, whereas vibratory compaction uses vibratory force to cause the asphalt mixture to undergo rotational movement, achieving a better compaction effect. Figure 6a,b represents the cloud diagram of the z-axis positions of model particles after each pass of static compaction and vibratory compaction. According to Figure 6a, each static compaction by the drum results in asymmetric wavy deformation of the pavement. This is due to the continuous static compaction action of the drum, which causes particles within the pavement to be unable to rotate effectively, leading to an asymmetric wavy deformation of the pavement. Especially after the first three static compactions, the asymmetric wave deformation of the model gradually diminishes as the static compaction pass increases. As shown in Figure 6b, the vibratory compaction of the drum also causes asymmetric wavy deformation of the pavement, but it is not as pronounced as with static compaction. In summary, during the compaction process, both static compaction and vibratory compaction can cause asymmetric wavy deformation of the pavement, but the former is more significant. This is because the high-frequency vibratory compaction of the asphalt mixture causes the mixture to rotate at a high frequency, effectively improving the asphalt pavement smoothness. Thus, for the same number of drum passes, vibratory compaction more effectively enhances pavement smoothness compared to static compaction.

### 4.2. Particle Angular Velocity

**Figure 7:**
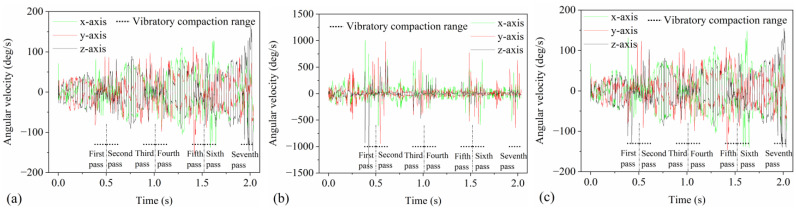
The changes in angular velocity over time during compaction: (**a**) mortar, (**b**) aggregate, and (**c**) mixture.

The angular velocities of three different types of particles—rotating around the x-axis (direction of drum rolling), y-axis (lateral direction of the pavement), and z-axis (thickness direction of the pavement)—change over time, as illustrated in Figure 7. Figure 7a–c represents mortar, aggregate, and mixture, respectively. To ensure the angular velocity’s validity, the average angular velocity of all particles of each type is used to represent that particle type’s angular velocity. As shown in Figure 7a, the angular velocity of the mortar in all three axes fluctuates during the static compaction process due to collisions and rotations caused by the static compaction of the drum. During the vibratory compaction process, the fluctuation frequency and amplitude of the mortar’s angular velocity increase significantly, indicating that the drum’s vibratory force enhances collisions and rotations among particles. As shown in Figure 7b, at the static compaction’s initial stage, the aggregate’s angular velocity fluctuates more significantly than the mixture’s, due to the aggregate’s loose arrangement, which leads to increased rotations and collisions among the aggregates. During vibratory compaction, the fluctuations in the angular velocity of the aggregate exceed those of the mixture and mortar, because the aggregates are larger and more irregular in shape, making their collisions and rotations more intense under the same vibratory force. As depicted in Figure 7c, the angular velocity of the mixture during the compaction period is slightly higher than that of the mortar, indicating that the rotational characteristics of the mixture are mainly influenced by the mortar, while the presence of aggregates increases the fluctuation amplitude of the mixture’s angular velocity. Therefore, angular velocity can reflect the dynamic rotational characteristics of particles in the asphalt pavement during the compaction process, aiding in understanding the mechanism of particle rotation during compaction from the particle perspective.

### 4.3. TAV of Particles

**Figure 8:**
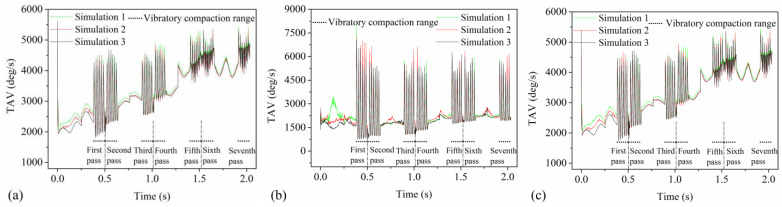
The changes in TAV over compaction time: (**a**) mortar, (**b**) aggregate, and (**c**) mixture.

The TAV of particles in the three models changes over compaction time as depicted in Figure 8, where particles in Figure 8a–c represent the mortar, aggregate, and mixture, respectively. The TAV, composed of angular velocities along three axes, signifies the overall magnitude of the rotational motion of particles in space. It can be represented by the Euclidean norm of the TAV vector (x, y, z), with the calculation formula as follows: $\sqrt{x^{2}+y^{2}+z^{2}}$. From Figure 8, it is evident that the TAVs in the three models change similarly but with differences in numerical fluctuations. These differences are due to the distinct distributions of aggregate and mortar in the models, causing unevenness in particle shape and mass distribution. From Figure 8a, it is observed that the mortar’s TAV increases with the compaction pass and eventually stabilizes, indicating that the rearrangement of particles and increased compaction limit the rotational movement of the particles. During the vibratory compaction process, the mortar’s TAV exhibits high-frequency fluctuations, suggesting the vibratory force induces rapid rotation and extensive rearrangement of particles. As shown in Figure 8b, the TAV of the aggregate remains relatively stable during static compaction indicating a smooth rotation under static compaction. In contrast, during vibratory compaction, the TAV of the aggregate shows high-frequency fluctuations, with the amplitude significantly exceeding that of the mixture and mortar. Figure 8c shows that the TAV of the mixture during compaction is similar to that of mortar, albeit with slightly lower values for the mortar. The specific reasons are the same as those discussed in Figure 7c. Therefore, the TAV of particles reflects the asphalt mixture particles’ evolution from a loose to a compact and stable state during compaction, serving as an important indicator for understanding asphalt pavements’ compaction mechanism.

### 4.4. Angular Velocity Amplitude of Asphalt Mixture

**Figure 9:**
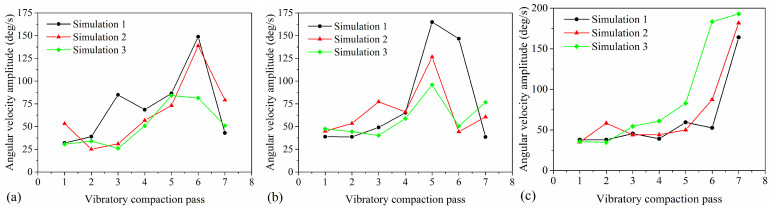
The change in angular velocity amplitude with vibratory compaction passes: (**a**) x-axis, (**b**) y-axis, and (**c**) z-axis.

To study the changes in the angular velocity of particles within the asphalt mixture during vibratory compaction, statistical analysis was conducted on the angular velocity of the mixture shown in Figure 7c. By calculating the average of all positive angular velocities and the average of all negative angular velocities during each pass of vibratory compaction, and then computing the difference between these two averages, the angular velocity amplitude for the mixture in that pass is obtained. Figure 9 shows the change in angular velocity amplitude across three axes (x, y, z) with the vibratory compaction pass, where Figure 9a–c corresponds to the particle rotation around the x-axis, y-axis, and z-axis, respectively. The moment of inertia, an important measure of the resistance to rotation, is calculated as the mass of the particle multiplied by the square of the distance from the particle to the axis of rotation. Hence, as the density between particles increases and they move closer to the rotation axis, the moment of inertia decreases. Moreover, according to the law of conservation of angular momentum (the product of moment of inertia and angular velocity), angular momentum remains constant when particles are subjected to a constant torque. With increased compaction, particles arrange more tightly, reducing the moment of inertia and thus increasing angular velocity. Figure 9a reveals that the angular velocity amplitude around the x-axis initially increases and then decreases, reaching a peak at the sixth pass. This is due to the dense arrangement of the mixture reducing the moment of inertia and thereby increasing the angular velocity. Subsequently, the formation of a dense structure among particles makes further compaction difficult, causing a decrease in angular velocity amplitude. Figure 9b shows that the change in angular velocity amplitude around the y-axis follows a similar pattern to the x-axis but peaks at the fifth pass. Therefore, the particle of asphalt pavement primarily rotates around the x-axis and y-axis (within the vertical plane of the pavement) in the first six passes of vibratory compaction. Figure 9c indicates that the angular velocity around the z-axis remains relatively stable before increasing, reaching its maximum at the seventh pass. This occurs as particles form a dense structure by the seventh pass, hindering further compaction and resulting in primary rotation around the z-axis (within the pavement’s horizontal plane). Hence, across the first six passes and at the seventh pass of vibratory compaction, asphalt mixtures predominantly rotate within the pavement’s vertical and horizontal planes, respectively. These findings provide a crucial perspective for understanding the compaction mechanism of asphalt mixtures.

### 4.5. TAV Amplitude

**Figure 10:**
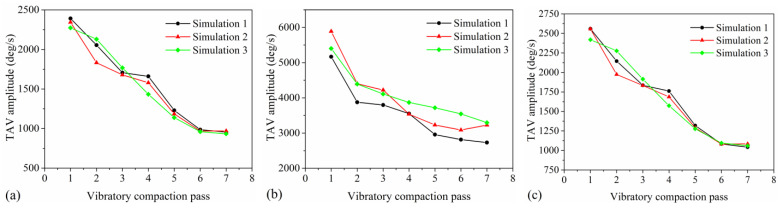
The changes in TAV amplitude with vibratory compaction passes: (**a**) mortar, (**b**) aggregate, and (**c**) mixture.

The TAV amplitude can quantitatively describe the rotational state of asphalt mixture particles in three-dimensional space. The TAV amplitude of the particles is obtained by calculating the average of all peak values and all trough values within each vibratory compaction pass, as depicted in Figure 8, and then determining the difference between these two averages. As the vibratory compaction pass increases, the changes in the TAV amplitude of the particles are depicted in Figure 10, with Figure 10a–c representing the changes in the mortar, aggregate, and mixture, respectively. The variation trend of the TAV amplitude of the three models with different types of particles in the process of vibration compaction is roughly the same, which indicates that the discrete element model of asphalt pavement is relatively stable. As seen in Figure 10a, it is evident that with the increase in vibration compaction passes, the TAV amplitude of the mortar shows a decreasing trend and finally tends to be stable. This suggests that friction and rearrangement among particles during the compaction process reduce their rotational kinetic energy in three-dimensional space, ultimately leading to stabilization. Figure 10b reveals that as compaction passes increase, the TAV amplitude of aggregates gradually decreases. Unlike the mixture, the reduction in rotational motion of aggregates is more significant due to their larger volume and inertia, which are more prominently affected during compaction. According to Figure 10c, the TAV change in the mixture mirrors that of the mortar but is slightly greater, attributed to the smaller size of mortar particles. The TAV amplitude reflects the enhanced interaction between particles in asphalt mixtures during compaction, leading to restrictions on particle rotation. Thus, it reveals that the three-dimensional dynamic rotation characteristics of particles are closely related to the compaction degree of the mixture, providing an effective indicator for the quantitative assessment of asphalt mixture compaction quality.

### 4.6. Correlation between TAV Amplitude and Compaction Degree

**Figure 11:**
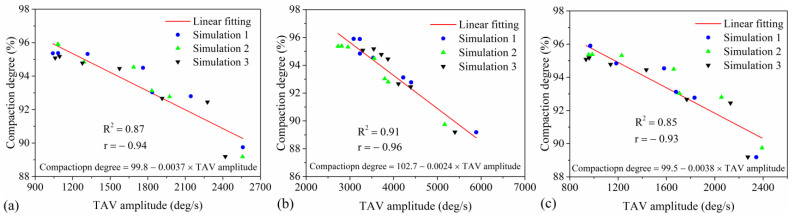
The correlation between TAV amplitude and compaction degree of the mixture: (**a**) mixture, (**b**) aggregate, and (**c**) mortar.

Figure 11 illustrates the correlation between TAV amplitude for three particle types—mixture, aggregate, and mortar—and the compaction degree of asphalt mixtures, with Figure 11a–c detailing each relationship, respectively. A linear fitting formula, revealing a linear correlation, was applied for a rapid quantitative assessment of the relationship between TAV amplitude and the compaction degree of the mixture. Figure 11 presents the fitting formula and correlation coefficients (R^2^) linking TAV amplitude to the compaction degree of the mixture. Figure 11a demonstrates a good correlation (R^2^ = 0.87) between the TAV amplitude of the mixture and its compaction degree. This suggests that TAV amplitude effectively predicts the compaction degree of asphalt mixtures, with the fitting formula assisting in stability evaluation under various compaction degrees. In Figure 11b, the aggregate particles exhibit a high correlation (R^2^ = 0.91), meaning the relationship between the TAV amplitude of aggregates and the compaction degree of the mixture is the most reliable. Consequently, the TAV amplitude of aggregates assesses their stability under varying compaction conditions and predicts the asphalt pavements’ compaction degree during compaction. Figure 11c shows a relatively reliable linear relationship (R^2^ = 0.85) for the mortar, indicating that the fitting formula for the mortar can predict its stability under different compaction degrees, thereby ensuring the compaction quality of the mortar. The analysis found a significant linear correlation between the TAV amplitude of the three types of particles and the compaction degree of the mixture, especially for aggregates (R^2^ = 0.91). Therefore, the TAV amplitude of aggregates can accurately predict the compaction degree of asphalt mixtures. The Pearson correlation coefficient (r) between the TAV amplitude of three particle types and the compaction degree of the mixture ranges from −0.93 to −0.96, indicating a very strong negative linear relationship. This demonstrates that TAV amplitude is a highly reliable predictor of mixture compaction degree. Utilizing the fitting formulas for the three particle types enables effective stability evaluation under varied compaction conditions. Overall, the linear relationship between the TAV amplitude and compaction degree of the mixture offers an effective method for controlling and predicting the compaction quality of asphalt pavements.

### 4.7. Particle CN

**Figure 12:**
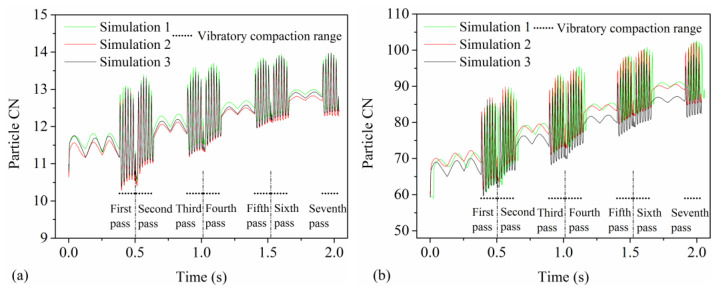
The changes in particle CN with compaction time: (**a**) mixture and (**b**) aggregate.

The particle CN represents the number of contacts a particle has with other particles at a specific time, reflecting the compactness and structural stability of the mixture. Figure 12a,b, respectively, shows the changes in particle CN for the mixture and the aggregate with compaction time during vibrational compaction. As shown in Figure 12a, the particle CN gradually increases with the increase in the static compaction pass of the drum, indicating the static compaction of the drum can uniformly enhance the compactness among mixture particles. Moreover, as the vibratory compaction pass increases, the fluctuation amplitude of the mixture’s particle CN decreases. This is due to the vibratory force of the drum effectively enhancing the changes in particle CN for the mixture and the aggregate, thereby promoting the formation of a more stable particle structure. Figure 12b shows that the changes in the CN of aggregates are similar to those of the mixture but with greater fluctuation amplitude during vibratory compaction. This indicates more active structural rearrangement among the aggregates, related to their larger size and irregular shape. Therefore, the particle CN can reflect the compactness and structural stability of asphalt pavements during compaction.

**Figure 13:**
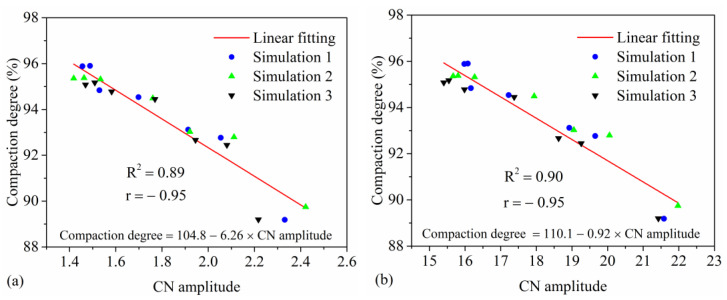
The correlation between CN amplitude and compaction degree of the mixture: (**a**) mixture and (**b**) aggregate.

The particle CN amplitude directly impacts the structural stability of asphalt pavements, while compaction degree is a critical parameter determining the load-bearing capacity and service life of asphalt pavements. The fluctuation pattern of the CN during vibratory compaction in Figure 12 is similar to the TAV shown in Figure 8. Therefore, the calculation method of the TAV amplitude from Figure 10 is employed to determine the CN amplitude. Figure 13a,b shows that there is a significant correlation between the CN amplitude of the mixture and aggregate and the compaction degree of the mixture, and the correlation coefficients are 0.89 and 0.90, respectively. The r for both the mixture and the aggregate is −0.95, indicating a strong and significant negative linear relationship between the CN amplitude and the compaction degree. Consequently, this demonstrates a highly reliable predictive relationship. The high correlation between the CN amplitude and mixture compaction degree reveals a close link between dynamic contact changes in particles and mixture density, providing a quantitative assessment method for the structural stability of asphalt pavements. Additionally, the fitting formula can help to predict the CN amplitude under different compaction degrees for the mixture or aggregates, thereby quantitatively assessing the structural stability of asphalt mixtures or aggregates.

## 5. Conclusions

This study utilized the DEM to construct a compaction model for the middle surface course of the asphalt pavement and verified its effectiveness. Initially, by monitoring the changes in the particle angular velocity and TAV of the mortar, aggregates, and mixture during compaction, the compaction mechanism of the asphalt pavement was preliminarily explained. Subsequently, the variations in the angular velocity amplitude of the mixture and the TAV amplitude of the mortar, aggregates, and mixture under different vibratory compaction passes were analyzed. Finally, the correlation between the TAV amplitude, the CN amplitude, and the compaction degree of the mixture was studied, respectively. The main conclusions are as follows:

- Both static and vibratory compaction cause asymmetric wave deformation in asphalt pavement, but vibratory compaction significantly reduces this deformation, enhancing pavement smoothness. This helps optimize compaction methods to enhance pavement smoothness, thereby improving driving comfort and durability.
- The particle angular velocity reflects the dynamic rotation of asphalt pavement particles during compaction, with mixtures primarily rotating within vertical planes during the first six passes and horizontal planes during the seventh pass. This helps to understand the rotation patterns of asphalt pavement particles during compaction.
- The TAV reflects the evolution of asphalt mixtures from a loose to a dense and stable state during compaction and reveals the three-dimensional dynamic rotational characteristics of particles. This provides crucial indicators for understanding the compaction mechanism of asphalt pavements.
- The TAV amplitude of the asphalt mixture, aggregates, and mortar all show a clear linear correlation with the mixture’s compaction degree, and their fitting formulas offer an effective quantitative method for controlling the compaction quality of asphalt pavements. This helps optimize the compaction process, ensuring the pavement achieves the desired density.
- The change in the particle CN and the close correlation between its amplitude and the compaction degree of the mixture provide an effective quantitative index for an in-depth understanding of the density of asphalt mixtures and the structural stability of aggregates. This helps monitor the stability of the aggregate structure during the compaction process of asphalt pavement courses.

This research explores the particle rotational motion characteristics of the middle surface course of asphalt pavement and their correlation with the compaction degree of the mixture. However, the relationship between different surface courses (top surface course, middle surface course, and bottom surface course) and particle motion (speed, angular velocity, and TAV) still requires further investigation. Therefore, it is hoped to promote further research to predict the compaction quality of asphalt pavements, thereby providing important theoretical support for achieving true “intelligent compaction“.

## Figures and Tables

**Table 1:** Technical specifications of SBS(I-D) modified asphalt.

Test Items	Values	Requirements	Methods (JTG E20-2011) [29]
Penetration (25 °C, 100 g, 5 s) (0.1 mm)	52	40~60	T0604-2011
Softening point (°C)	73.0	≥60	T0606-2011
Ductility (5 °C, 5 cm/min) (cm)	36	≥20	T0605-2011
Density (15 °C) (g/cm^3^)	1.031	Measured value	T0603-2011

**Table 2:** Basic technical specifications of aggregates.

Aggregate	Test Items	Value	Requirement	Methods (JTG E42-2005) [30]
Coarse	Crushing value (%)	12.5	≤24	T0316-2005
	Apparent density (g/m^3^)	2.714	≥2.50	T0304-2005
	Water absorption rate (%)	0.18	≤3.0	T0304-2005
	Soft stone content (%)	2.9	≤5	T0320-2005
	Los Angeles wear loss (%)	7.3	≤30	T0317-2005
Fine	Apparent relative density (Fine) (g/m^3^)	2.711	≥2.50	T0328-2005
	Water absorption rate (Fine) (%)	0.37	≤3.0	T0330-2005

**Table 3:** The material physical parameters.

Type	Poisson’s Ratio	Density (kg/m^3^)	Young’s Modulus (MPa)
Aggregate	0.275	2902	60,000
Mortar	0.4	2510	4

**Table 4:** The contact model parameters.

Contact Interface	Constant Pull-Off Force (N)	Surface Energy (J/m^2^)	Contact Plasticity Ratio	Slope Exponent	Tensile Exponent	Tangential Stiffness Multiplier
Aggregate and aggregate	0	0	0	1	1	0.294
Aggregate and mortar	−0.02	100	0.8	1.5	1	0.281
Mortar and mortar	−0.02	100	0.8	1.5	1	0.267

**Table 5:** The compaction process of the roller at the experimental site.

Compaction Pass	Driving Mode	Front Drum	Rear Drum
Precompaction	Forward driving	Static compaction	Static compaction
First/Third/Fifth/Seventh pass	Backward driving	Static compaction	Vibratory compaction
Second/Fourth/Sixth pass	Forward driving	Vibratory compaction	Static compaction

**Table 6:** The relative error for different compaction passes.

Compaction Pass	−1	0	1	2	3	4	5	6	7
Experimental compaction degree (%)	85.40	88.83	90.40	92.43	93.03	94.13	95.10	95.00	94.80
Simulated compaction degree (%)	85.15	88.64	89.38	92.67	92.94	94.49	94.98	95.48	95.44
Relative error (%)	−0.29	−0.22	−1.13	0.26	−0.10	0.38	−0.13	0.51	0.68

## Data Availability

Data are contained within the article.
